# Genes, environments, and traits: Can you predict when we are going to understand them?

**DOI:** 10.3389/fgene.2026.1979650

**Published:** 2026-10-30

**Authors:** José M. Álvarez-Castro

**Affiliations:** Department of Education, University and Professional Training, Xunta de Galicia, Santiago de Compostela, Spain

**Keywords:** environmental factors, explanation, gene mapping, NOIA, polygenic risk score, prediction

## Abstract

As genomics has entered the ‘big data’ era over the past 2 decades, phenotypic prediction has become central to fields ranging from livestock production to human health. In the latest case, efforts have focused primarily on the development of polygenic risk scores (PRS), derived from genome-wide association (GWA) studies. As prediction methods gained popularity, earlier gene-mapping approaches have been largely perceived as a dead end. Gene-mapping experiments did not rapidly reach the high level of expectation they raised, and the incentives for further improving their methodologies soon decreased. It could be argued that the study of genotype-phenotype relationships has taken sides in the broader scientific debate between prediction and explanation (also referred as causation or understanding). But fortunately, a major obstacle to the search of complex, polygenic (or even more broadly, polyfactorial) bases of traits has recently been overcome. Technically, the Natural and Orthogonal InterActions (NOIA) model enables unbiased estimates of effects of complex genetic and environmental architectures of traits, even in datasets with severe departures from equilibrium genotypic frequencies. This achievement significantly increases our potential to reveal the factors underlying phenotypes and allows for a sounder interpretation and reliable comparisons of different mapping experiments on the same trait. Improving our understanding of the relationship among genes, environments, and traits may provide us with countless advantages. Thus, the potential of advanced genetic modeling for studying complex traits needs to be assessed in practice, particularly when evidence suggests that PRS may be approaching a structural limit.

## Introduction

Since the explosion of genomic data, the quantitative genetics community has devoted a vast amount of resources to predicting trait values from panels of genetic markers (see, e.g., Ahmandi and Bartholomé, 2022). Originally, a major expectation of this line of work was to bypass the expenses and time-consuming procedures required to phenotype bulls for the dairy cattle industry. In line with the classic selection-index framework for combining information from multiple traits (Hazel and Lush, 1942), Meuwissen et al. (2001) accounted for (genome-wide) information from multiple markers to perform accurate breeding value predictions. Their goal was to enable selection of young bulls before the approximately 5-year progeny-testing period required to obtain daughter records. However, this method could not completely substitute phenotyping because it was necessary to continually incorporate newly phenotyped animals into the reference population in order to avoid a steady decline in accuracy over generations (e.g., Pszczola and Calus, 2016).

The aforementioned statistical framework for genomic prediction was soon extended to human genetics with minor modification. Wray et al. (2007) developed the theoretical basis for predicting individual disease susceptibility from genome-wide association (GWA) data, and the International Schizophrenia Consortium (2009) proposed that combining the effects of large numbers of common genetic variants into a single index could predict disease risk in specific individuals. These studies established the foundation for what are now known as polygenic risk score (PRS) indexes, which have since become a widespread tool in genetics applied to human health for estimating inherited susceptibility to complex diseases among individuals within a given population.

Thus, phenotypic prediction from genomic data has became well established in current quantitative genetics analyses applied to both agriculture, livestock production, and human genetics (Arirangan et al., 2026). In this communication, we advocate that it is convenient to also devote significant efforts to better understanding the genetic and environmental architectures of traits. The high expectations put on quantitative trait loci (QTL) analyses were not achieved in the short time and that was used as a justification to switch the focus towards prediction (e.g., Hill, 2010). Thus, the real potential of gene-mapping to disclose the genetic and environmental factors underlying traits has remained largely unexplored. Moreover, a new window of opportunity has been open to address that task (Álvarez-Castro, 2023), as we further develop below. Before that, we briefly review what can be expected from PRS.

## The reach of PRS

PRS are used to quantify genetic predisposition to complex traits and diseases by aggregating the effects of genetic variants identified through genome-wide association (GWA) studies (e.g., Ma and Zhou, 2021). The ability to stratify individuals according to inherited risk was improved and PRS have shown potential applications in biomedical research, disease prevention, and personalized medicine (e.g., Inouye et al., 2018; Kullo et al., 2022). However, their predictive performance varies across traits and populations, while population diversity, disease prevalence, integration with non-genetic risk factors, amongst other issues, may condition clinical implementation in practice (Martin et al., 2019).

Moreover, recent analyses suggest that PRS predictive accuracy is approaching a plateau for many complex traits, with diminishing returns from increasing sample sizes of GWA studies alone (Henches et al., 2025). Indeed, PRS accuracy has increased rapidly since the emergence of large-scale GWA studies, but the rate of improvement associated with more recent increases in sample sizes of GWA studies has decreased substantially, suggesting diminishing returns from expanding discovery cohorts. Then, Henches et al.’s (2025) analyses point to the need of additional strategies such as improved variant coverage through whole-genome sequencing and more comprehensive modeling of genetic variation.

Such structural limitations of PRS should not come as a surprise, for two reasons. On the one hand, while a simple model may be used to build a convenient predictive tool, it must not be viewed as a detailed explanation of a complex reality. The prediction strategy in quantitative genetics relies on overly simplified assumptions for the underlying models (as, e.g., Hill, 2014 explicitly assumes) as a way to be able to handle enormous datasets. GWA studies have been interpreted to equate with prediction (e.g., Goddard et al., 2009), but the assumption of purely additive gene action is unrealistic, inherently limiting the predictive fit that GWA studies can achieve.

On the other hand, the strategy of prediction methodologies like genomic prediction and PRS is to use information from many markers at a time. Maximizing the inclusion of informative genetic signals implies relaxing statistical selection criteria and thus entails a trade-off between retaining genuine associations and excluding spurious ones (e.g., Herzig et al., 2022). This argument applies not only to additive effects but also to interactions, when the latter are taken into account. Thus, by construction, overfitting brings in a noisy effect, which can be shrunk but not completely removed. Overall, we are just at the verge when the conceptual limitations of PRS as a method of prediction are becoming apparent in practice.

## Elusive genetic architectures

In the context of quantitative genetics and evolutionary biology, Tautz et al. (2026) have recently highlighted the importance of uncovering the complex genetic and environmental factors underlying trait variation. In reality, their claim has long been a central focus of the field (see Govindaraju et al., 2026), and it is a key assumption when analyzing core evolutionary phenomena such as evolvability (the capacity for adaptive evolution; see Hansen et al., 2023). Tautz et al. (2026) have particularly criticized the approach to the understanding of trait determination by trying to disclose and interpret its underlying genes one by one, i.e., the aim to make use of individual QTL. This criticism was also raised by those supporting the switch toward prediction (Hill, 2010). QTL analysis was in fact originally set on the evaluation of likelihood-of-odds (LOD) scores calculated at each position in the genome by comparing the likelihood of a QTL at that position with the likelihood under the null hypothesis of no QTL effect (Lander and Botstein, 1989). The extension of this basic principle to assuming the action of more than one QTL in the same chromosomal region was soon addressed (Zeng, 1993), but it suffered from intricate difficulties (Zou and Zeng, 2008).

The difficulties of gene-mapping arise in practice in two seemingly opposing ways. On the one hand, low effects would require extremely large sample sizes to be detected at a statistically significant level, which leads to false negatives. Furthermore, even large effects have been difficult to detect statistically in populations that depart from ideal equilibrium frequencies and/or when allele substitutions affects a trait in different directions depending on the genetic background (e.g., Yang, 2004; Zeng et al., 2005; Wang and Zeng, 2006). On the other hand, false positives are also a concern since the early description of ghost QTL (Martínez and Curnow, 1992). In GWA studies, false positives may arise due to population structure, which frequently generates correlations in allele frequencies among even physically unlinked markers (e.g., Stephens and Balding, 2009; Coop et al., 2010). More to the point, it has been shown that departures from linkage equilibrium may lead to the detection of unreal interaction effects in gene mapping experiments (Zan et al., 2018).

Since the advent of QTL analysis, considerable progress has been made in different fields that significantly improved gene-mapping procedures. From molecular tools enabling the routine use of dense marker maps, through statistical methods, to computing power, mapping experiments (including their spin-off towards GWA studies) advanced light-years beyond their early stages in a relatively short period of time (e.g., Doerge, 2002; Mackay et al., 2009). However, serious risks of both false positives and false negatives remained in the studies, which understandably may have raised more than reasonable doubts about the convenience of continuing to devote resources to improving genetic mapping tools.

## New prospects for understanding

The initial steps of QTL detection were implemented with the models of genetic effects then available—those stemming from the very origins of quantitative genetics (Fisher, 1918; Cockerham, 1954; Kempthorne, 1954). Those models had been developed under the paradigm of prediction (e.g., Falconer and MacKay, 1996) and were poorly suited for the paradigm of explanation of evolutionary phenomena (e.g., Hansen, 2013). It is indeed well known that models suited for these two paradigms differ radically (Shmueli, 2010). Overall, it became clear that models of genetic effects, at the core of the detection and estimation of effects in gene mapping, needed to be improved (e.g., Yang, 2004; Zeng et al., 2005; Álvarez-Castro and Carlborg, 2007; Álvarez-Castro and Yang, 2011; Ma et al., 2012).

Although resolving this issue took longer than implementing the improvements discussed in the previous section, doing so has opened up new opportunities for more efficient and reliable gene mapping. Indeed, although the two obstacles to genetic mapping also mentioned in the previous section work in opposite directions, both have defective genetic modeling as a common underlying cause (as further commented below). This is why the development of a model of genetic and environmental effects that fits the paradigm of understanding and explanation—the natural and orthogonal interactions (NOIA) model—has made it possible to help overcome both problems (Álvarez-Castro, 2023).

Models of genetic effects provide a framework for describing phenotypes and phenotypic variation in terms of genetic and environmental influences and their interactions. The precision and generality of these models determine the extent to which the properties of genetic architectures and environmental contributions can be understood, as well as the efficiency of gene-mapping studies. The key advantages of NOIA over alternative models are outlined below.

First, from its original setting (Álvarez-Castro and Carlborg, 2007), NOIA unifies under the same mathematical framework formulations of genetic effects under both a population and an individual perspective, thus enabling all kinds of biological interpretations of complex genetic and environmental underlying factors. Second, NOIA embraces a set of developments enabling researchers to deal with complex genetic architectures, including (taken together, if necessary) multiple multiallelic autosomal and/or sex-linked and/or sex-dependent loci and multiple multifactorial environmental variables, with arbitrary values of dominance, parent-of-origin and epistatic interactions, as well as gene-environment and environment-environment interactions (Álvarez-Castro and Yang, 2011; Álvarez-Castro, 2014; Álvarez-Castro, 2020; Álvarez-Castro, 2023).

And third, the population-oriented effects as implemented in NOIA retain both the proper biological interpretation and the required statistical properties (like orthogonality) in the face of arbitrary departures of the genotypic frequencies from ideal equilibrium situations like Hardy-Weinberg proportions and linkage equilibrium, as well as from random associations between/among genes and environments (Álvarez-Castro and Crujeiras, 2019; Álvarez-Castro, 2020; Álvarez-Castro, 2023). This latest advantage is key to address both limitations raised in the previous section. Indeed, despite working in apparently opposite directions (generating both false negatives and false positives), they have in common that they are typically caused by estimates of effects being biased by population facts not properly accounted for by the models (e.g., Yang, 2004; Zeng et al., 2005; Wang and Zeng, 2006). The latest version of NOIA (Álvarez-Castro, 2023) does not only overcome this problem of biased estimations, but it does so under the assumption of as complex as desired genetic architectures (as mentioned above as second advantage), and even increases biological interpretation with population-independent formulations of genetic and environmental effects (as mentioned as first advantage).

In short, the conceptual limitations of the prediction strategies that have switched the inertia of quantitative genetics away from gene mapping seem to start becoming evident in practice, right at the time when more holistic approaches aiming to address causation are being encouraged, and NOIA enhances our ability to pursue such approaches. The benefits of deepening our understanding of the biology underlying traits of interest would undoubtedly be rewarding, even in unpredictable ways. In this context, revitalizing gene mapping need not come at the expense of prediction-based approaches, but could instead foster a fruitful complementarity, potentially giving rise to synergies between the two.

## Discussion

Plomin and von Stumm (2022) explicitly addressed the debate of prediction versus understanding (which they specifically referred to as explanation) in human genetics. While they stressed the need of the first, they do not neglect that a parallel line of work may be kept on running to try and achieve understanding of biological mechanisms in the long term. Incidentally, we argue that the time is ripe to seriously reevaluate the extent to which recently achieved advanced genetic and environmental modeling may overcome methodological hurdles to disclose, replicate, and interpret the genetic and environmental bases of complex traits.

It is worth emphasizing at this point that it is in the deepest sense of this expression that we propose to examine the factors underlying complex traits. This is to say, it looks sensible to assume that the genetic and environmental basis of traits will in many cases, if not all, be intrigued, implying various genes (or even more generally, loci, including, e.g., regulatory regions) and environmental factors and different kinds of (within gene, gene-gene, gene-environment, and environment-environment) interactions. Having into account the methodologies initially available, it looks reasonable having at first followed the strategy of working out a gene at a time. However, resolving the doubt of to what extent genetic architectures may actually be disclosed requires the use of a sound multifactorial approach.

Prediction not only is a valued scientific achievement from the philosophy of science viewpoint (e.g., Shmueli, 2010), but it was really the only way possible to proceed at the origin of quantitative genetics, when abstract Mendelian factors were acknowledged to underlie continuous variation and gradual evolution (Fisher, 1918), but would only be soundly assigned to a physical status one generation later (Hershey and Chase, 1952; Franklin and Gosling, 1953; Watson and Crick, 1953). Nowadays, genetic trait prediction using molecular methods is undeniably convenient and practical. However, this does not mean wiping gene mapping off the map. Likewise, strong criticism on the initial gene-by-gene discovery strategy is well-founded, but it is not adequate justification for abandoning gene mapping.

In 2007, NOIA was proposed as a unifying theory with a mathematical structure showing practical advantages towards addressing general genetic architectures in arbitrary populations (Álvarez-Castro and Carlborg, 2007). At that time, a huge amount of resources was being allocated to prediction in quantitative genetics. Had more resources been devoted to complete the development of NOIA, it could have by now already served the aim of causation by assessing the extent to which genetic/environmental architectures of traits can be disclosed without the drawback of estimation biases. Notably, even when NOIA was not yet fully developed to aid causation, understanding and explanation, it has already been used mostly to assess and improve prediction (Chevalet et al., 2010; Toro and Varona, 2010; Duenk et al., 2020; Wientjes et al., 2022; see Álvarez-Castro, 2023). Apropos, NOIA having been satisfactorily applied to genomic prediction can be interpreted as a validation that the difficulties that will naturally arise when implementing NOIA in gene mapping shall just as well be overcome.

In essence, NOIA currently brings within reach applications with the potential to overcome limitations that have traditionally constrained the pursuit of the complex basis underlying traits. There is no doubt that a better understanding of causation in genetics could yield far‐reaching benefits across a wide range of fields, particularly genetic epidemiology. Therefore, we are hereby breaking a lance for that to happen.

## References

[B1] Ahmandi N. Bartholomé J. (2022). Genomic Prediction of Complex Traits, Methods and Protocols. New York: Humana.

[B2] Álvarez-Castro J. M. (2014). Dissecting genetic effects with imprinting. Front. Ecol. Evol. 2, 51. 10.3389/fevo.2014.00051 PMC4259164 25583081

[B3] Álvarez-Castro J. M. (2020). Gene-environment interaction in the era of precision medicine - filling the potholes rather than starting to build a new road. Front. Genet. 11, 921. 10.3389/fgene.2020.00921 33133127 PMC7575001

[B4] Álvarez-Castro J. M. (2023). Genes, Environments and Interactions. Evolutionary and Quantitative Genetics Brought up-to-date. Cham, Switzerland: Springer.

[B5] Álvarez-Castro J. M. Carlborg Ö. (2007). A unified model for functional and statistical epistasis and its application in quantitative trait loci analysis. Genetics 176, 1151–1167. 10.1534/genetics.106.067348 17409082 PMC1894581

[B6] Álvarez-Castro J. M. Crujeiras R. M. (2019). Orthogonal decomposition of the genetic variance for epistatic traits under linkage disequilibrium-applications to the analysis of bateson-dobzhansky-muller incompatibilities and sign epistasis. Front. Genet. 10, 54. 10.3389/fgene.2019.00054 30891057 PMC6411799

[B7] Álvarez-Castro J. M. Yang R.-C. (2011). Multiallelic models of genetic effects and variance decomposition in non-equilibrium populations. Genetica 139, 1119–1134. 10.1007/s10709-011-9614-9 22068562 PMC3247674

[B8] Arirangan S. De Oliveira L. F. Hasan M. N. A B. S. Tuinstra M. Brito L. F. (2026). Sharing approaches in predictive genomics across animals, plants and humans. Nat. Genet. 58, 503–516. 10.1038/s41588-025-02491-w 41639408

[B9] Chevalet C. Servin B. Sancristobal M. (2010). “Including non-additive effects in Bayesian methods for the prediction of genetic values from genome-wide SNP data,” in *10th WCGALP*: German Society of Animal Science.

[B10] Cockerham C. C. (1954). An extension of the concept of partitioning hereditary variance for analysis of covariances among relatives when epistasis is present. Genetics 39, 859–882. 10.1093/genetics/39.6.859 17247525 PMC1209694

[B11] Coop G. Witonsky D. Di Rienzo A. Pritchard J. K. (2010). Using environmental correlations to identify loci underlying local adaptation. Genetics 185, 1411–1423. 10.1534/genetics.110.114819 20516501 PMC2927766

[B12] Doerge R. W. (2002). Mapping and analysis of quantitative trait loci in experimental populations. Nat. Rev. Genet. 3, 43–52. 10.1038/nrg703 11823790

[B13] Duenk P. Bijma P. Calus M. P. L. Wientjes Y. C. J. Van Der Werf J. H. J. (2020). The impact of non-additive effects on the genetic correlation between populations. G3 (Bethesda) 10, 783–795. 10.1534/g3.119.400663 31857332 PMC7003072

[B14] Falconer D. S. Mackay T. F. C. (1996). Introduction to Quantitative Genetics. Harlow: Prentice Hall.

[B15] Fisher R. A. (1918). The correlation between relatives on the supposition of Mendelian inheritance. Trans. Roy. Soc. Edinb. 52, 339–433. 10.1017/S0080456800012163

[B16] Franklin R. Gosling R. G. (1953). Molecular configuration in sodium thymonucleate. Nature 171, 740–741. 10.1038/171740a0 13054694

[B17] Goddard M. E. Wray N. R. Verbyla K. Visscher P. M. (2009). Estimating effects and making predictions from genome-wide marker data. Stat. Sci. 24, 517–529. 10.1214/09-sts306

[B18] Govindaraju D. R. Álvarez-Castro J. M. Mallet J. Templeton A. R. Townsend J. P. Edwards S. V. (2026). Locating Lewontin on the genotype-phenotype map. Genetics iyag275. 10.1093/genetics/iyag275 42933027

[B19] Hansen T. F. (2013). Why epistasis is important for selection and adaptation. Evolution 67, 3501–3511. 10.1111/evo.12214 24299403

[B20] Hansen T. F. Houle D. Pavlicev M. Pélabon C. (2023). in Evolvability: A Unifying Concept in Evolutionary Biology (Cambridge, London: The MIT Press).

[B21] Hazel L. N. Lush J. L. (1942). The efficiency of three methods of selection. J. Hered. 33, 393–399. 10.1093/oxfordjournals.jhered.a105102

[B22] Henches L. Kim J. Yang Z. Rubinacci S. Pires G. Albinana C. (2025). Polygenic risk score prediction accuracy convergence. HGG Adv. 6, 100457. 10.1016/j.xhgg.2025.100457 40375557 PMC12167061

[B23] Hershey A. D. Chase M. (1952). Independent functions of viral protein and nucleic acid in growth of bacteriophage. J. Gen. Physiol. 36, 39–56. 10.1085/jgp.36.1.39 12981234 PMC2147348

[B24] Herzig A. F. Clerget-Darpoux F. Genin E. (2022). The false dawn of polygenic risk scores for human disease prediction. J. Pers. Med. 12, 1266. 10.3390/jpm12081266 36013215 PMC9409868

[B25] Hill W. G. (2010). Understanding and using quantitative genetic variation. Philos. Trans. R. Soc. Lond B Biol. Sci. 365, 73–85. 10.1098/rstb.2009.0203 20008387 PMC2842708

[B26] Hill W. G. (2014). Applications of population genetics to animal breeding, from wright, fisher and lush to genomic prediction. Genetics 196, 1–16. 10.1534/genetics.112.147850 24395822 PMC3872177

[B27] Inouye M. Abraham G. Nelson C. P. Wood A. M. Sweeting M. J. Dudbridge F. (2018). Genomic risk prediction of coronary artery disease in 480,000 adults: implications for primary prevention. J. Am. Coll. Cardiol. 72, 1883–1893. 10.1016/j.jacc.2018.07.079 30309464 PMC6176870

[B28] Kempthorne O. (1954). The correlation between relatives in a random mating population. Proc. R. Soc. Lond. B. Biol. Sci. 143, 102–113. 10.1098/rspb.1954.0056 13224653

[B29] Kullo I. J. Lewis C. M. Inouye M. Martin A. R. Ripatti S. Chatterjee N. (2022). Polygenic scores in biomedical research. Nat. Rev. Genet. 23, 524–532. 10.1038/s41576-022-00470-z 35354965 PMC9391275

[B30] Lander E. S. Botstein D. (1989). Mapping mendelian factors underlying quantitative traits using RFLP linkage maps. Genetics 121, 185–199. 10.1093/genetics/121.1.185 2563713 PMC1203601

[B31] Ma Y. Zhou X. (2021). Genetic prediction of complex traits with polygenic scores: a statistical review. Trends Genet. 37, 995–1011. 10.1016/j.tig.2021.06.004 34243982 PMC8511058

[B32] Ma J. Xiao F. Xiong M. Andrew A. S. Brenner H. Duell E. J. (2012). Natural and orthogonal interaction framework for modeling gene-environment interactions with application to lung cancer. Hum. Hered. 73, 185–194. 10.1159/000339906 22889990 PMC3534768

[B33] Mackay T. F. Stone E. A. Ayroles J. F. (2009). The genetics of quantitative traits: challenges and prospects. Nat. Rev. Genet. 10, 565–577. 10.1038/nrg2612 19584810

[B34] Martin A. R. Kanai M. Kamatani Y. Okada Y. Neale B. M. Daly M. J. (2019). Clinical use of current polygenic risk scores may exacerbate health disparities. Nat. Genet. 51, 584–591. 10.1038/s41588-019-0379-x 30926966 PMC6563838

[B35] Martínez O. Curnow R. N. (1992). Estimating the locations and the sizes of the effects of quantitative trait loci using flanking markers. Theor. Appl. Genet. 85, 480–488. 10.1007/BF00222330 24197463

[B36] Meuwissen T. H. Hayes B. J. Goddard M. E. (2001). Prediction of total genetic value using genome-wide dense marker maps. Genetics 157, 1819–1829. 10.1093/genetics/157.4.1819 11290733 PMC1461589

[B37] Plomin R. Von Stumm S. (2022). Polygenic scores: prediction versus explanation. Mol. Qsyciatry 27, 49–52. 10.1038/s41380-021-01348-y 34686768 PMC8960389

[B38] Pszczola M. Calus M. P. (2016). Updating the reference population to achieve constant genomic prediction reliability across generations. Animal 10, 1018–1024. 10.1017/S1751731115002785 26711815

[B39] Schizophrenia_Consortium I. (2009). Common polygenic variation contributes to risk of schizophrenia and bipolar disorder. Nature 460, 748–752. 10.1038/nature08185 19571811 PMC3912837

[B40] Shmueli G. (2010). To explain or to predict? Stat. Sci. 25, 229–310. 10.1214/10-sts330

[B41] Stephens M. Balding D. J. (2009). Bayesian statistical methods for genetic association studies. Nat. Rev. Genet. 10, 681–690. 10.1038/nrg2615 19763151

[B42] Tautz D. Pallares L. F. Andersson L. Barghi N. Barton N. Bay R. (2026). Beyond mendel: a call to revisit the genotype-phenotype map through new experimental paradigms. Genetics 232, iyag024. 10.1093/genetics/iyag024 41701356 PMC13050187

[B43] Toro M. A. Varona L. (2010). A note on mate allocation for dominance handling in genomic selection. Genet. Sel. Evol. 42, 33. 10.1186/1297-9686-42-33 20699012 PMC2928189

[B44] Wang T. Zeng Z. B. (2006). Models and partition of variance for quantitative trait loci with epistasis and linkage disequilibrium. BMC Genet. 7, 9. 10.1186/1471-2156-7-9 16472377 PMC1456990

[B45] Watson J. D. Crick F. H. C. (1953). A structure for deoxyribose nucleic acid. Nature 171, 737–738. 10.1038/171737a0 13054692

[B46] Wientjes Y. C. J. Bijma P. Calus M. P. L. Zwaan B. J. Vitezica Z. G. Van Den Heuvel J. (2022). The long-term effects of genomic selection: 1. Response to selection, additive genetic variance, and genetic architecture. Genet. Sel. Evol. 54, 19. 10.1186/s12711-022-00709-7 35255802 PMC8900405

[B47] Wray N. R. Goddard M. E. Visscher P. M. (2007). Prediction of individual genetic risk to disease from genome-wide association studies. Genome Res. 17, 1520–1528. 10.1101/gr.6665407 17785532 PMC1987352

[B48] Yang R.-C. (2004). Epistasis of quantitative trait loci under different gene action models. Genetics 167, 1493–1505. 10.1534/genetics.103.020016 15280257 PMC1470938

[B49] Zan Y. Forsberg S. K. G. Carlborg O. (2018). On the relationship between high-order linkage disequilibrium and epistasis. G3 (Bethesda) 8, 2817–2824. 10.1534/g3.118.200513 29945968 PMC6071592

[B50] Zeng Z. B. (1993). Theoretical basis for separation of multiple linked gene effects in mapping quantitative trait loci. Proc. Natl. Acad. Sci. U. S. A. 90, 10972–10976. 10.1073/pnas.90.23.10972 8248199 PMC47903

[B51] Zeng Z. B. Wang T. Zou W. (2005). Modeling quantitative trait loci and interpretation of models. Genetics 169, 1711–1725. 10.1534/genetics.104.035857 15654105 PMC1449562

[B52] Zou W. Zeng Z. B. (2008). Statistical methods for mapping multiple QTL. Int. J. Plant Genomics 2008, 286561. 10.1155/2008/286561 18551187 PMC2423007

